# Characteristics Assessment of Online YouTube Videos on Radiotherapy for Lung Cancer

**DOI:** 10.7759/cureus.19150

**Published:** 2021-10-30

**Authors:** Jim (Zhang Hao) Li, Meredith Giuliani, Paris-Ann Ingledew

**Affiliations:** 1 Department of Surgery, Division of Radiation Oncology, University of British Columbia, Faculty of Medicine, Vancouver, CAN; 2 Department of Radiation Oncology, University of Toronto, Toronto, CAN

**Keywords:** online health information, youtube videos, radiotherapy (rt), lung cancer, patient education

## Abstract

Introduction

The internet has become a mainstay source of health information for cancer patients. Online patient education videos are common; however, there have been no studies examining the quality of publicly available videos on radiotherapy for lung cancer (one of the most common forms of cancer). To fill this knowledge gap, we aim to systematically map and objectively assess videos discussing radiotherapy for lung cancer on YouTube.

Methods

The terms “radiotherapy for lung cancer,” “radiation for lung cancer,” “radiation therapy for lung cancer,” and “radiation treatment for lung cancer” were searched on YouTube using a clear-cache browser. Results were sorted by relevance and the top 50 English-language results for each search were recorded. After removing duplicates, each video was assessed for length, Video Power Index (VPI, which is the product of a video’s average daily views and like and dislike ratio), source, content, comment moderation, and misinformation. Two raters were used to ensure consistency. Results were evaluated using descriptive and inferential statistics.

Results

A total of 88 unique videos resulted from the search. The median video length was 4 minutes and 5 seconds. The average VPI was 10.9 (95% CI: 1.5-20.4) and the median number of views was 954.5. All videos were published between July 8, 2009 and November 18, 2020. Of the videos, 44% were published within the past two years. A total of 61% of the videos were from the USA, 14% were from the UK, 6% from Australia, 5% each from Canada and India, and other countries make up the remaining 10%. Most of the videos were published by healthcare facilities (39%) and non-profit organizations (31%). Content-wise, 95% of videos contain information specific to lung cancer. A total of 46 videos (52%) were targeted toward patient education. Of which, 37 covered radiotherapy for lung cancer, 12 covered side effects for radiotherapy, and 11 covered both. The other 42 videos (48%) were designed for a professional audience. Stereotactic body radiation therapy (SBRT)/stereotactic ablative radiotherapy (SABR) was the most commonly described radiotherapy modality (42%), and the physician interview was the most common format, being used in 59% of videos. Out of the 38 videos with at least one comment, only two (5%) were moderated by the host channel. None of the videos featured misleading information.

Conclusions

This study comprehensively surveyed YouTube videos pertaining to radiotherapy for lung cancer to provide a high-level overview of the information that patients may find online. Although nearly half of the videos describe lung cancer radiotherapy for patients, only a small proportion comprehensively cover both radiotherapy and its side effects. The results of our study can help guide the development of patient education tools and encourage healthcare providers to recognize the limitations of online health information and proactively address patient questions regarding radiotherapy. Future research could examine videos on other lung cancer treatment options or radiotherapy for other cancers.

## Introduction

Lung cancer is the leading cause of cancer deaths worldwide and ranks the second-highest in incidence [1]. Currently, radiation therapy (RT) is one of the main modalities of lung cancer treatment, as it offers a survival benefit for patients at various stages of small cell lung cancer (SCLC) and non-small cell lung cancer (NSCLC) [2,3]. However, not all patients who are eligible receive it. It has been estimated that 64% of NSCLC patients require RT, but in practice, only 32% of NSCLC patients in the United States receive RT as part of their treatment [2,4]. This proportion is even smaller in low-income countries, where less than 10% of eligible patients receive RT [5]. Besides lack of insurance and treatment availability, other commonly cited barriers to receiving RT include lack of information and inaccurate perceptions regarding RT, its efficacy, and side effects [6-8]. Access to reliable and accurate information is therefore critical to ensuring that patients can receive RT when indicated.

To illustrate the role of online health information, 80% of internet users are seeking health information online, a trend that has been increasing in recent years [9,10]. In particular, YouTube is becoming more prominent as a platform for disseminating health information [11]. It is the second most visited website in the world, where users may search for medical information using its large, diverse, and free-to-access video library [12]. Although research on personal health can be empowering for patients [13], a seminal 2012 systematic review revealed concerns regarding the prevalence of misleading information and lack of content regulation on YouTube [11]. This in turn creates potentially harmful situations where patients receive conflicting information, which could result in delays or refusal of necessary interventions such as RT for lung cancer. Previous research has shown that only 19.3% of text-based websites discussing adverse events of RT for lung cancer were considered high-quality as per the Health on the Net Foundation Code of Conduct (HONcode) framework and that this trend is consistent across multiple languages [14]. Other recent studies examining the quality of YouTube videos for other cancers have generally demonstrated considerable space for improvement and concluded that YouTube is insufficient on its own to provide reliable patient education [15,16]. However, to the best of our knowledge, there are no specific studies examining the quality of YouTube videos regarding RT for lung cancer. This indicates a gap in the literature, which we will attempt to address through conducting this study.

## Materials and methods

Extracting videos

The terms “radiotherapy for lung cancer,” “radiation for lung cancer,” “radiation therapy for lung cancer,” and “radiation treatment for lung cancer” were inputted into the YouTube search engine. The results were sorted by “Relevance,” and the top 50 results for each search were recorded, with the aim of capturing all videos that patients would have reasonably been exposed to. After removing duplicates, we screened all results against pre-determined inclusion criteria: videos must be (a) currently accessible (i.e. not scheduled for future release), (b) not behind a paywall, and (c) in the English language. A rather lenient set of inclusion criteria was employed since the primary purpose of this research was to objectively characterize what resources are available to patients. To minimize undue influences from the researcher’s geographical location and search history, all searches were performed using a clear-cache Google Chrome browser (Google Inc., Mountain View, CA) in incognito mode.

Characteristics assessment

To the best of our knowledge, there is no comprehensive standard pre-existing tool for assessing online videos in the patient education setting [17]. Although traditional tools exist for evaluating the quality of online patient education materials, such as the Health on the Net (HON) Foundation code [18], the DISCERN scale [19], and the Journal of American Medical Association (JAMA) benchmarks [20], they are not specifically designed for videos. As such, rather than focus on an evaluation of quality, for the purposes of this study, we developed a systematic approach to collect objective video parameters. The approach was divided into three broad domains: general parameters, video source, and video content. Within the general parameters domain, each video was assessed for its number of views, date of publication, number of likes and dislikes, Video Power Index (VPI, which is the product of a video’s average daily views and like and dislike ratio) [21], and video length. It should be noted that VPI is a commonly used parameter in similar research to objectively measure video popularity by taking into account the average number of views per day and the like and dislike ratio, but it has no bearing on quality [21]. The video source domain specified each video’s country of origin, publisher, and presenter(s). Finally, the video content domain included topic(s) covered, media type, comment moderation, advertisements, and gross misinformation. These parameters were selected as they correlated or were analogous to previously validated markers used to characterize health information in traditional media such as books or websites [18-20]. For example, the “publisher” and “presenter(s)” are analogous to disclosing authorship as per the JAMA benchmarks [20]. Similar approaches have been employed by other studies examining the content of online health videos - common metrics include the number of views, like and dislike ratio, video length, publisher, and topic(s) covered [22-24]. After data collection, results were evaluated using descriptive statistics (i.e., measures of central tendency) and inferential statistics (i.e., chi-square test) using the statistical software R, version 3.6.1 (R Foundation, Vienna, Austria).

Inter-rater reliability

The reliability of the video descriptions was confirmed via consensus between two independent assessments by a radiation oncologist with lung cancer expertise and a medical student. A number was assigned to each video and a random number generator was used to select a random sample of 10 videos for two researchers to evaluate independently by applying the characterization approach. Inter-rater reliability was highly consistent among both raters; for every category, a kappa value or intraclass correlation coefficient >0.70 was achieved, thereby indicating good inter-rater agreement. Any discrepancies were resolved by discussion and the remaining videos were then assessed by one researcher independently.

## Results

General parameters

The top 50 YouTube results for the search terms “radiotherapy for lung cancer,” “radiation for lung cancer,” “radiation therapy for lung cancer,” and “radiation treatment for lung cancer” were compiled. After applying the exclusion criteria and removing all duplicates, 88 unique videos remained.

**Figure 1:**
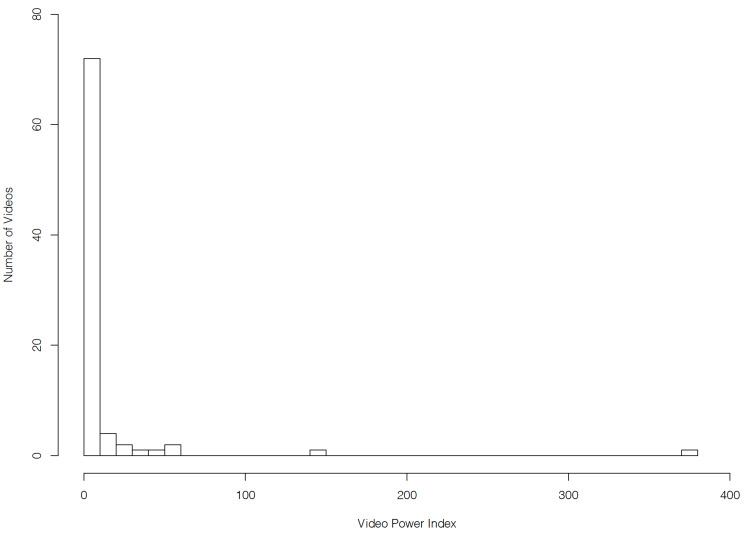
Histogram of the Video Power Index of YouTube videos describing radiotherapy for lung cancer.

The popularity of YouTube videos covering radiotherapy for lung cancer followed a positively skewed distribution. Although the mean number of views per video was 25,487, the median was only 955. The range varied from 46 to 1,228,659 views. It should be noted that the video with 1,228,659 views received more views than all the other 87 videos combined. The newest video was published 52 days before the time of the search, while the oldest was already published for 4,203 days. A total of 69% of videos were more than two years old and 57% were more than three years old. The Video Power Index (VPI) was calculated by multiplying a video’s average daily views by its like and dislike ratio [21]. Similar to the number of views, the VPI also followed a positively skewed distribution, with a mean of 10.9 and a median of 1.0 (Figure 1). VPI ranged from 0.05 to 376. The median video length was 4 minutes and 5 seconds. Videos from commercial channels had a median length of 2 minutes and 11 seconds while those from non-commercial channels had a median length of 4 minutes and 32 seconds.

Video source

Most videos originated from the USA (61%), followed by the UK (14%), Australia (6%), Canada (5%), and India (5%). The remaining 10% were from other countries (Table 1). The publisher channels were categorized into healthcare, non-profits, commercial, education, and personal. Most videos were from either a healthcare (39%) or non-profit (31%) publisher. A total of 18% were from commercial sources, 9% from educational channels, and 3% from personal accounts (Table 1). Information was presented by a physician and/or allied health workers in 77% of videos, while information was presented by a patient in 18% of videos. Non-profit publishers were more likely to feature a patient presenter compared to other publisher categories (P = 0.014).

**Table 1:** Compiled categorical results of YouTube videos on radiotherapy for lung cancer. * Resultant categorical variables not mutually exclusive with each other.

Parameter	Results	Number of videos (percent of total)
Date of video publication	<2 years ago	27 (31)
	2-3 years ago	11 (12)
	>3 years ago	50 (57)
Video source	USA	54 (61)
	UK	12 (14)
	Australia	5 (6)
	Canada	4 (5)
	India	4 (5)
	France	2 (2)
	The Netherlands	2 (2)
	Other countries	5 (6)
Publisher affiliation	Healthcare facility	34 (39)
	Non-profit	27 (31)
	Commercial	16 (18)
	Educational institution	8 (9)
	Personal	3 (3)
Media type/video format*	Physician interview	52 (59)
	Video tours	20 (23)
	Patient interviews	19 (22)
	Lecture-style presentations	17 (19)
	Computer graphics	11 (13)
Radiotherapy modality*	Stereotactic body radiation therapy	37 (42)
	Intensity-modulated radiation therapy	11 (13)
	Proton therapy	9 (10)
Comment moderation	Present	2 (2)
	Absent	36 (41)
	No comments seen	30 (34)
	Comments disabled	20 (23)

Video content

**Figure 2:**
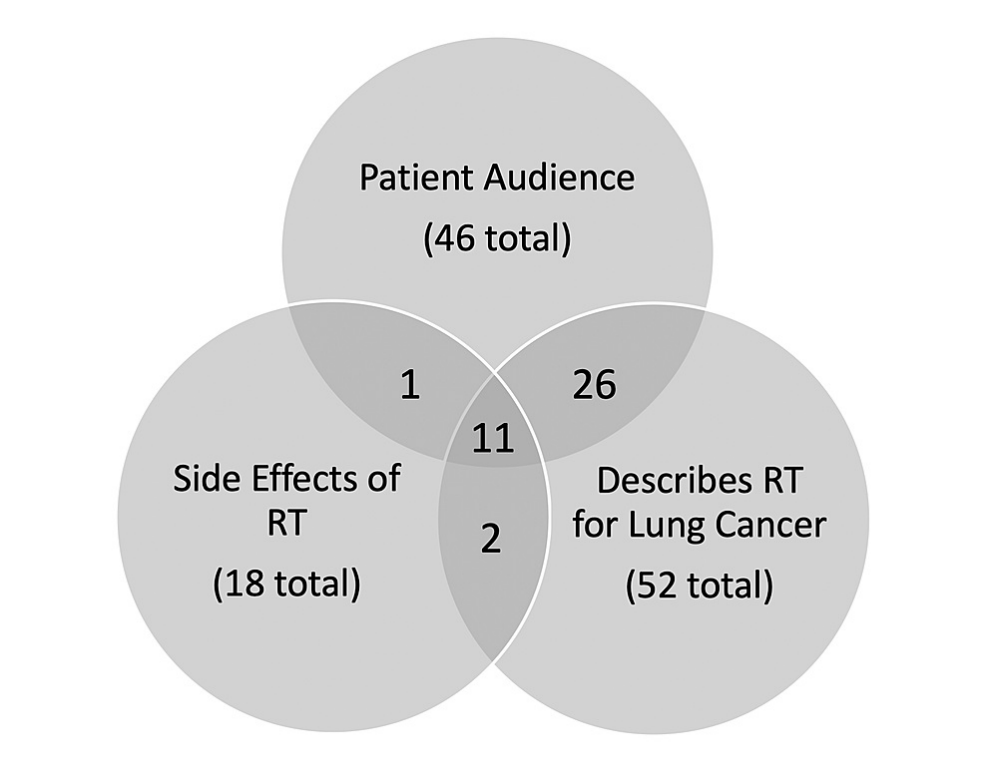
Venn diagram of the number of videos intended for patient education, describing radiotherapy for lung cancer, and explaining the side effects of radiotherapy. RT = radiation therapy.

Of the 88 included videos, 46 (52%) were subjectively assessed to be targeted toward a patient audience. The other 42 (48%) were designed for health professionals. Although 84 videos (95%) presented information related to lung cancer, only 52 described radiation therapy (RT) for its treatment, and 18 covered the side effects of RT. In total, 11 videos (13%) were directed toward a patient audience while adequately describing RT for lung cancer and its side effects (Figure 2).

The physician interview was the most commonly used format, being featured in 59% of videos, followed by live video tours (23%), patient interviews (22%), lecture-style presentations (19%), and computer graphics/animations (13%) (Table 1). Some videos employed multiple formats. In terms of RT modalities, as previously mentioned, only 52 (59%) videos described at least one modality. Stereotactic body radiation therapy (SBRT) was mentioned by 37 videos (42% of total), followed by intensity-modulated radiation therapy (IMRT) at 13% and proton therapy at 10% (Table 1). Again, multiple modalities could be described in a single video. Comments were enabled in 68 videos. Of these, 38 had at least one comment. However, only two videos’ comment sections were moderated by the publisher, as evidenced by them responding to at least one viewer comment (Table 1). Advertisements were seen in six videos (7%). None of the videos featured grossly inaccurate or misleading information.

## Discussion

Out of the 88 videos selected for characterization, only 27 were published within the past two years, and the majority (50 videos) were published more than three years ago. This raises a point of concern as patients may not necessarily be receiving up-to-date information about treatment options. This is consistent with existing literature that suggests inadequate currency among online cancer information [25]. We acknowledge that our search was filtered by “Relevance” instead of “Upload Date,” where the latter would show the newest videos on top. Therefore, currency rates may be higher had we performed the search by “Upload Date.” However, since “Relevance” is the default setting, we believe using this would be most representative of what patients would practically encounter. Nevertheless, our findings still indicate that YouTube alone is inconsistent in delivering up-to-date information and that healthcare professionals will need to continue informing patients about the standard of care.

Videos offer an advantage over traditional text-based education materials as they are less prone to poor readability hindering patient comprehension, and there is evidence to suggest superior knowledge retention [26]. To illustrate the effect that video format has on quality, a 2019 study on thyroid cancer videos showed that animated videos were not statistically different from non-animated videos in terms of length, VPI, and quality [15], while a more recent study by Kuru and Erken in 2020 suggested that quality may be higher in videos presented by physicians as opposed to non-physicians [27]. In the context of our study, it is encouraging that the physician interview was the most commonly used format, and we would further encourage physicians to guide patients to videos produced by other healthcare professionals.

Commercialization of online health information is associated with lower information quality and a higher risk of featuring biased information [28]. However, as none of the videos in our study featured grossly inaccurate or misleading information, there is insufficient statistical power for us to compare between videos from commercial versus non-commercial sources. Interestingly, previous literature has suggested that objective and subjective measures of video quality are positively correlated to video length [15,23,24]. Combined with our study findings that the median length of commercial videos was shorter than their non-commercial counterparts, it is possible that a discrepancy in quality favoring non-commercial videos would exist as well. However, as our goal was not to assess video quality, this remains speculative.

With regards to the target audience, there is roughly an even distribution of videos targeted toward patients and healthcare professionals (46 versus 42). Although the “signal-to-noise” ratio may seem poor at a glance, it is reasonable to assume that most patients would skim the titles and thumbnails of the first few videos to find a video appropriate for them. A greater concern lies within video content - out of the 46 videos targeted toward a patient audience, only 11 described both what radiotherapy is and its side effects when used for cancer treatment (Figure 2). Since side effects of treatment is a topic of high patient interest [29], many patients may be left with insufficient information to guide their decision-making. Therefore, it is imperative that healthcare professionals take the time to specifically discuss details such as side effects and direct patients to appropriate resources.

Another interesting finding is the lack of visible comment moderation on the vast majority of included videos. Out of the 38 videos with at least one comment, only two comment sections were moderated by the host channel via the presence of replies. We acknowledge that some channels may moderate comments by deleting undesired content. However, it is not possible for the researchers to determine if any comments have been deleted, therefore host replies were used as a proxy. Another 30 videos had no comments. Considering the low rate of comment moderation in the videos with comments, it is unlikely that a large portion of those 30 videos would have moderated comment sections. The remaining 20 videos disabled comments altogether. The near-complete absence of comment moderation means that misinformation - either intentional or unintentional - could be easily spread among viewers even if the video itself does not. This is especially pertinent for videos with a high view count. Although disabling comments may seemingly prevent the spread of misinformation, it also precludes constructive viewer interactions. Previous research has shown that comment sections can serve as a platform for collaborative deliberation, which in turn stimulates higher levels of knowledge construction among readers [30]. Especially for sensitive topics such as lung cancer, having a safe platform where patients can share their experiences, support each other, and offer constructive feedback to the channel can be of immense benefit.

Limitations to our study include the fact that it only represents a snapshot in time. The YouTube algorithm for displaying videos may change over time, and videos’ view counts will increase at different rates, which further alter their visibility. In addition, due to resource constraints, our search was limited to the English language. In the future, analyses may be performed for searches in additional languages, and video characteristics may be compared to determine if a difference exists between videos in various languages. Most importantly, as an objective characteristics assessment, we did not attempt to assess videos for quality. A direction for future research would be to analyze video quality for multiple cancers and to compare them against each other.

## Conclusions

Most YouTube videos describing radiotherapy for lung cancer are delivered by physicians and published by non-commercial sources. However, the currency is inconsistent, comment sections are poorly moderated, and nearly half of the videos are not targeted toward a patient audience. In addition, only a small proportion of the videos aimed toward a patient audience comprehensively cover both radiotherapy and its side effects. Nevertheless, despite the limitations of YouTube videos describing radiotherapy for lung cancer, healthcare professionals should recognize that online videos could still be a powerful form of patient education and empowerment. Not only does it circumvent traditional barriers such as reading comprehension, but it also offers the opportunity to deliver high-quality information in an era where telemedicine is becoming increasingly prominent. As it stands, there is a vast amount of unfulfilled potential that could be better harnessed to complement the patient-physician relationship in advancing patient-centered decision-making.
